# Insignificant Impact of Chemotactic Responses of *Pseudomonas aeruginosa* on the Bacterial Attachment to Organic Pre-Conditioned RO Membranes

**DOI:** 10.3390/membranes9120162

**Published:** 2019-12-02

**Authors:** Lan Hee Kim, Johannes S. Vrouwenvelder

**Affiliations:** 1 Water Desalination and Reuse Center (WDRC), Division of Biological and Environmental Science and Engineering (BESE), King Abdullah University of Science and Technology (KAUST), Thuwal 23955-6900, Saudi Arabia; lanhee.kim@kaust.edu.sa; 2 Department of Biotechnology, Faculty of Applied Sciences, Delft University of Technology, Van der Maasweg 9, 2629 HZ Delft, The Netherlands

**Keywords:** conditioning film, chemotaxis, *Pseudomonas aeruginosa* PAO1, reverse osmosis membrane

## Abstract

We investigated the impact of conditioning compositions on the way bacteria move and adhere to reverse osmosis (RO) membranes that have been pre-conditioned by organic compounds. We used humic acid (HA), bovine serum albumin (BSA), and sodium alginate (SA) to simulate conditioning layers on the RO membranes. First, we investigated the chemotactic responses of *Pseudomonas aeruginosa* PAO1 to the organic substances and the impact of changes in physicochemical characteristics of pre-conditioned membranes on bacterial attachment. Second, we observed bacterial attachment under the presence or absence of nutrients or microbial metabolic activity. Results showed that there was no relationship between the chemotactic response of *P. aeruginosa* PAO1 and the organic substances, and the changes in hydrophobicity, surface free energy, and surface charge resulting from changing the composition of the conditioning layer did not seem to affect bacterial attachment, whereas changing the roughness of the conditioned membrane exponentially did (exponential correlation coefficient, *R*^2^ = 0.85). We found that the initial bacterial attachment on the membrane surface is influenced by (i) the nutrients in the feed solution and (ii) the microbial metabolic activity, whereas the chemotaxis response has a negligible impact. This study would help to establish a suitable strategy to manage bacterial attachment.

## 1. Introduction

Membrane fouling in membrane-based water treatment systems negatively affects the membrane’s performance by significantly reducing the water flux and reducing the lifetime of the membrane [1,2]. Membrane fouling is formed by adsorption or deposition of organic, inorganic, particulate/colloidal, and biological matters on the membrane surfaces [1]. The formation of a biofilm on the surface of the membrane (biofouling) is considered to be a significant problem due to the growth of bacteria, even after a pretreatment process that removes 99.9% of bacteria [1,3]. The formation of biofilms on the surface of a membrane occurs in different stages: (i) the awareness and transport of the bacteria to the surface, (ii) the reversible adhesion of the bacterial cells to the membrane, (iii) the irreversible attachment of bacteria to surface and the development of a biofilm due to bacterial growth and the production of extracellular polymeric substances (EPS) [1,2,4]. The irreversible bacterial attachment is mediated by the physicochemical properties of the substratum and the surface properties of bacterial cells, and system operational conditions (hydrodynamics, bulk solution properties) [5].

In this irreversible initial bacterial attachment stage, a “conditioning film” is formed by the deposition or absorption of natural organic matter (NOM) and EPS in natural water on the membrane’s surface [6]. The conditioning film can either improve or impair the initial bacterial attachment on the surface of the membrane by changing of membrane characteristics such as hydrophobicity, surface charge, and roughness, based on the composition of deposited organic matters resulting from the different physicochemical interactions between the conditioning film layer (formed by substratum) and the bacteria [6,7]. The impact of the organic compound-conditioning layer on the reverse osmosis (RO) or the nanofiltration (NF) membrane surface properties such as surface charge, hydrophobicity, and topography on the bacterial attachment has been extensively studied (Table 1) [8,9,10,11,12,13,14].

Several studies have found that the conditioning film could improve bacterial attachment and the development of biofilms [8,13,15,16]. Subramani et al. (2009) reported that the increase of the membrane’s roughness by bovine serum albumin (BSA) or alginic acid (AA) enhanced bacterial attachment on NF and RO membranes [8]. Furthermore, the presence of biopolymers and humic substances on the membrane related directly to the rate of biofilm development [15]. Additionally, the presence of a conditioning film on the RO membrane caused more bacterial attachment and a rapid decline of the flux, in comparison with a clean membrane [13,16]. On the other hand, other studies reported that changes in membrane properties by the conditioning film inhibited attachment of bacteria [9,11]. Zhao et al. (2015) showed that the changes of conditioned NF membranes to the smooth, hydrophilic, highly negative surface charges by sodium alginate (SA) and humic acid (HA)-calcium conditioning layers inhibited bacterial attachment [9]. Heffernan et al. (2014) showed that bacterial attachment was inhibited by HA or AA conditioning layers on an NF membrane due to the requirement of energy for bacteria to penetrate the conditioning layer [11]. A better understanding of the various physicochemical properties of the biofouling layer, according to the type of conditioning of the film, would help to develop new biofouling control strategies for pretreatment methods or membrane modification.

Bacteria can move from bulk fluid to the surface of the membrane by Brownian motion, sedimentation, or convective mass transport [17]. Once bacteria are close to the surface, they are propelled either randomly (e.g., by a stream of fluid flowing over a surface) or in a directed fashion via chemotaxis and motility [18]. Chemotaxis, the process by which bacterial cells migrate toward favorable chemicals and away from unfavorable ones, is crucial for their survival and growth in natural environments [19].

Although research has been carried out on the impact of the conditioning layer on bacterial attachment and the chemotaxis response to the surface [8,9,10,11,12,13,14], no study has been done on the relationship between the conditioning layer and the chemotaxis response of bacteria, although this is a critical aspect to consider when studying biofouling and the impact of the conditioning layer. This has motivated us to study (i) the relationship between the bacterial chemotaxis response and the composition of the conditioning film, (ii) the impact of physicochemical properties of (HA, BSA, SA)-conditioned RO membranes on bacterial attachment, (iii) the impacts of nutrients in the feed solution, and (iv) the bacterial metabolic activity on the bacterial attachment (Table 2). In the work reported here, the conditioning layers on the RO membrane were formed by the organic substances such as HA, BSA, and SA (or Xanthan Gum, XG). The chemotactic response of *P. aeruginosa* PAO1 to the organic substances and swarming motility were investigated as well as the impact of the nutrients in the feed solution, microbial activity, and membrane-surface properties on the bacterial attachment.

## 2. Materials and Methods

### 2.1. Chemotaxis Responses of P. aeruginosa PAO1 to the Organic Substances in the Feed Solution

Chemotaxis responses of *P. aeruginosa* PAO1 were investigated by following Adler’s capillary method [20]. The model organic compounds, HA (Sigma Aldrich, St. Louis, MO, USA), BSA (Sigma Aldrich, USA), and XG (as an acidic polysaccharide) (Sigma Aldrich, USA) were used as chemoattractants, at concentrations of 0.05, 0.1, 0.5, 1, 50, and 100 mg/L, respectively. A total of 20 µL of solution for each chemoattractant was added in the capillaries; one end of the capillaries was blocked to create a vacuum condition. Capillaries containing each chemoattractant were put in the 1.5 mL microtube containing *P. aeruginosa* PAO1 cultures (10^6^ cells/mL) and the capillaries were taken out from the microtube after 1 h reaction at room temperature (RT; 25 ± 1 °C) (*n* = 4). A total of 20 µL of the solution (containing chemoattractants and transported bacterial cells) from the capillaries were mixed with 80 µL of phosphate-buffered saline (PBS; Life Technologies, Carlsbad, CA, USA) to make a total volume of 100 µL for additional cell number measurement. The same procedures were repeated for each organic compound used. The bacterial cells were stained with SYTO9 (Invitrogen, Carlsbad, CA, USA) and the total number of cells was determined using a BD Accuri^TM^ C6 flow cytometer (BD, Franklin Lakes, NJ, USA). The chemotaxis response ratio was obtained by calculating the ratio between the total number of cells in the capillaries containing the chemoattractant and the total number of cells in the capillaries without any chemoattractant (deionized (DI) water) (Equation (1)).

(1) $$\mathrm{Chemotaxis}\;\mathrm{response}\;\mathrm{ratio}\;=\;\frac{\mathrm{Total}\;\mathrm{cell}\;\mathrm{numbers}\;\mathrm{in}\;\mathrm{capillary}\;\mathrm{with}\;\mathrm{chemoattractant}}{\mathrm{Total}\;\mathrm{cell}\;\mathrm{numbers}\;\mathrm{in}\;\mathrm{capillary}\;\mathrm{without}\;\mathrm{chemoattractant}}$$

To analyze the surface charge of suspended organic substances, 100 mg/L of alginate, BSA, HA, and XG solutions were prepared in DI water and the surface charge was analyzed using a zetasizer Nano ZS (Malvern, Malvern, UK). The pH of solutions was ranged from 6.5 to 8.0.

### 2.2. Swarming Behavior of P. aeruginosa PAO1 on Swarm Plates

Semi-solid agar was prepared using a mixture of 1% peptone, 0.5% NaCl and 0.25% Bacto-agar [21]. A total of 100 mg/L of BSA, SA (Sigma-Aldrich, USA), and HA were dropped on a cellulose filter paper (2.5 µm pore size, Whatman, Maidstone, UK) and allowed to diffuse from the membrane into the semi-solid media for 16 h at RT. *P. aeruginosa* PAO1 cells were incubated in an Luria–Bertani (LB) medium (BD, USA), for 16 h at 30 °C. A total of 10 µL of cultured bacteria (10^5^ cells/mL) were inoculated at the center of the plate. The tip with the bacteria solution was inserted into the agar and ejected bacteria solution when the tip was pulled up through the media [21]. After inoculation, the agars were incubated at 30 °C, for 24 h. The diameter of the distance moved from the edge of the membranes was measured and averaged (*n* = 7).

### 2.3. Impact of Physicochemical Properties of Organic Pre-Conditioned Membranes on Bacterial Attachment

Conditioning film layers on brackish water reverse osmosis (RO) membrane coupons (DOW Chemicals, Midland, MI, USA) were formed by placing the RO membrane coupons into three different solutions containing 100 mg/L of either HA, BSA, or SA in DI water; solutions were subsequently incubated for 16 h at RT. The pre-conditioned membranes were dried at 37 °C for 24 h and the surface of the membrane was analyzed using an Agilent 5500 SPM atomic force microscopy (AFM; Agilent, Santa Clara, CA, USA) in tapping mode, and with a silicon cantilever (FESPA-V2; BRUKER, Billerica, MA, USA). The membrane’s roughness (*S_q_*) and surface’s skewness (*S_sk_*) were calculated by analysis of the AFM images, using Gwyddion software (version 2.47, Brno, Czech Republic). The surface free energy of conditioned membrane coupons was analyzed by contact angle (KRUSS, Hamburg, Germany) with two polar liquids (DI water, formamide), and one non-polar liquid (diiodomethane), as diagnostic liquids, respectively [22]. The surface free energy (*γ_s_*) was determined using the Owens–Wendt method based on the sum of a polar (*γ_s_^p^*) and dispersion (*γ_s_^d^*) components. The surface charge (zeta potential) of the conditioned membrane coupons was analyzed by SurPass^TM^ Electrokinetic analyzer (Anton Paar, Graz, Austria) with 10 mM NaCl, for pH ranging from 3 to 7. The zeta potential was calculated from the streaming potential value, using the Helmholz–Smolukowski (HS) equation [23].

### 2.4. Impact of Nutrients in the Feed Solution on Bacterial Attachment

**Figure 1:**
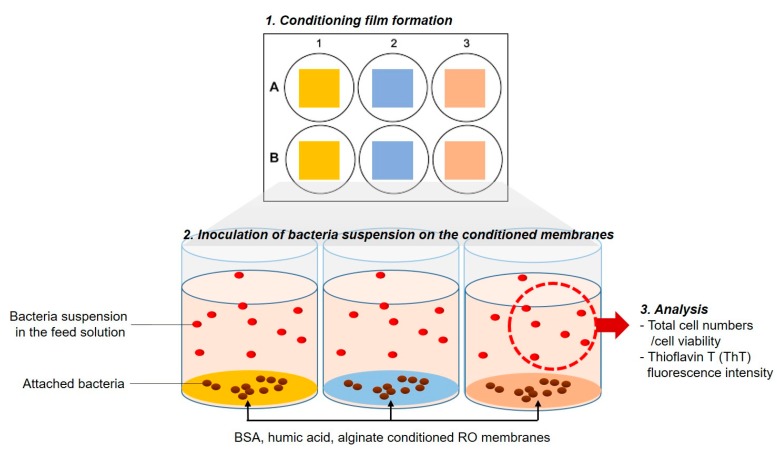
Schematic diagram of bacterial attachment experimental procedures. (1) Formation of the conditioning films on the reverse osmosis (RO) membrane in the 6-well microplates, (2) inoculation of *P. aeruginosa* PAO1 bacterial cells in each well, (3) analysis of the total number of bacteria cells, cell viability, and bacterial thioflavin T (ThT) fluorescence intensity in the feed solution.

In order to assess the role of the conditioning film composition on bacterial attachment, we investigated the physiological responses of *P. aeruginosa* PAO1 to organic substances, using either a solution that contained a nutrient or a solution with no nutrients at all. Bacterial cells were incubated in an LB medium and stained using a thioflavin T (ThT; Sigma-Aldrich, USA) staining dye at a final concentration of 10 µM. For the feed solution with no nutrients, cultured bacteria in the LB medium were washed three times with PBS, and optical density was adjusted at 600 nm (OD_600_) to 1.0. The conditioning film on the RO membrane was prepared using membrane coupons of 2 × 2 cm^2^ soaked in 100 mg/L of BSA, HA, AA, and incubated for 16 h at RT. The conditioned membranes were subsequently washed 3 times with PBS and attached on 6-well microplates. For the feed solution with no nutrients (especially no carbon source), 8 mL of PBS and 8 µL of a bacteria solution (OD_600_ 1.0) were injected into the microplate. For the feed solution containing a nutrient, 8 mL of M9 medium (Table S1) and 8 µL of bacteria solution (OD_600_ 1.0) were injected. The microplate was incubated at 30 °C and 90 rpm, and samples of suspension (suspended bacterial cells) and membrane coupons were taken at 6 h of incubation. To determine the total number of cells, the cell viability, and the bacterial motility, 1 mL of suspension samples was stained using the Live/Dead BacLight bacterial viability kit (Molecular Probes, Eugene, OR, USA) and 10 µM of ThT fluorescence dye, respectively (Figure 1). At each sampling time, membranes were taken out and dried at 37 °C for 24 h to measure the membrane’s roughness, using AFM (Agilent 5500 SPM, USA).

### 2.5. Impact of Microbial Activity in the Feed Solution on Bacterial Attachment

*P. aeruginosa* PAO1 was incubated in an LB broth (BD, USA) for 16 h. The cultured cells were collected by centrifugation at 8000 rpm for 10 min and subsequently washed 2 times with a PBS buffer. A total of 2.5% of glutaraldehyde was added to the washed cells (OD_600_, 1.0) and incubated for 2 h, at RT, following the protocol described in [24]. Cells were washed 2 times with a PBS buffer, and the total number of cells was determined using a BD Accuri^TM^ C6 flow cytometer (BD, USA).

The organic substance-conditioned RO membrane coupons (2 × 2 cm^2^) were placed in the 6-well microplate, to which we added 8 mL of PBS. Then, 8 µL of glutaraldehyde-treated or untreated *P. aeruginosa* PAO1 bacterial cells were injected into the conditioned membranes and placed in a 6-well microplate. The microplate was subsequently incubated at 30 °C and 90 rpm and took suspension samples at 0 h and 6 h. Total cell numbers were measured using BD Accuri^TM^ C6 flow cytometer (BD, USA).

### 2.6. Statistics Analysis

The *p* value was calculated by performing a two-tail student’s *t*-test using a GraphPad prism 6.0 (Graph Pad Software, San Diego, CA, USA). The obtained value was below 0.05 (*p* ≤ 0.05), which is considered to be a significant difference (*p* > 0.05 is considered non-significant).

## 3. Results and Discussion

### 3.1. Strong Chemotactic Responses of P. aeruginosa PAO1 to the HA in the Feed Solution

**Figure 2:**
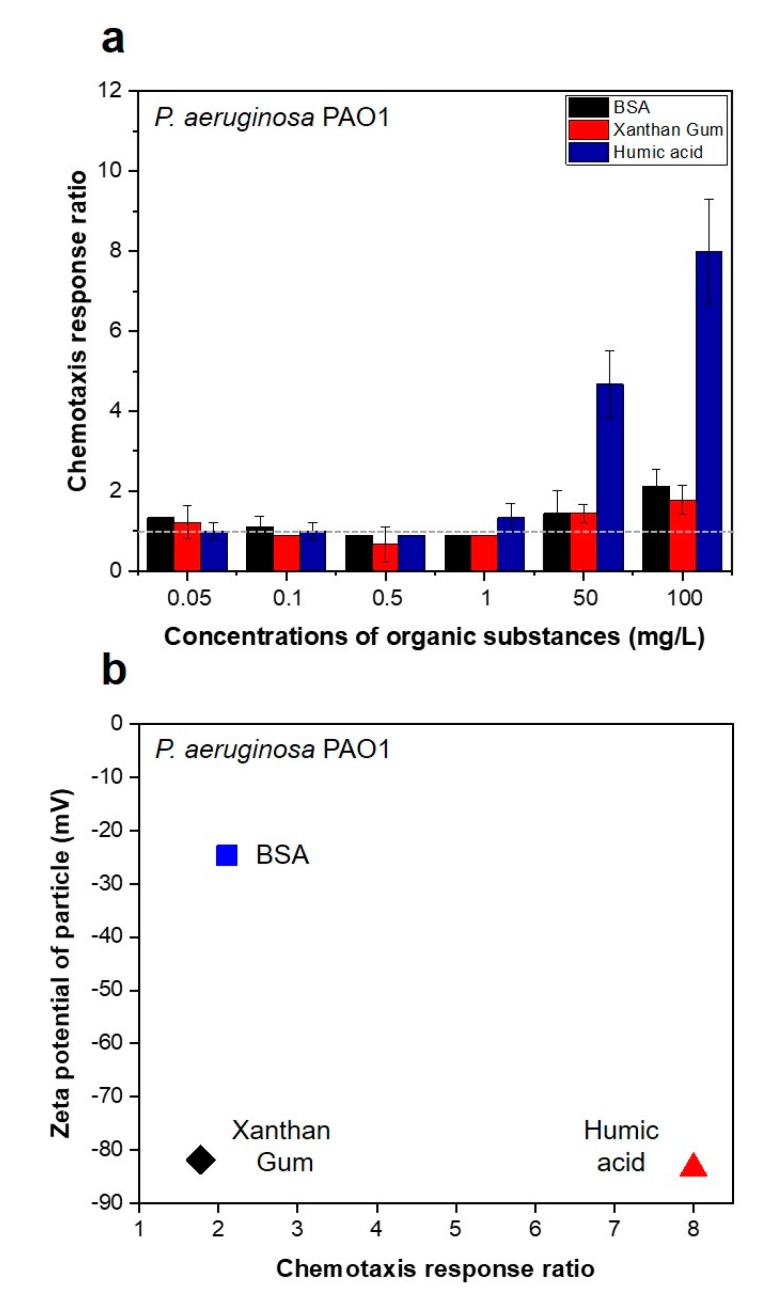
(**a**) Ratio of chemotaxis response of *P. aeruginosa* PAO1 to bovine serum albumin (BSA), xanthan gum (XG), and humic acid (HA) in solution. (**b**) Relationship between the surface charge of organic substances and the chemotaxis response ratio of *P. aeruginosa* PAO1.

Figure 2 shows the chemotaxis responses of *P. aeruginosa* PAO1 to the organic substances in the capillary systems, including the HA, BSA, and XG in the feed solution. A response ratio of “1” indicates no chemotactic response to the chemoattractant (HA, BSA, XG, in this study). A chemotaxis response ratio above 1 was obtained, when using 50 mg/L of BSA and XG, and 1 mg/L of HA with *P. aeruginosa* PAO1. HA strongly attracted *P. aeruginosa* PAO1, with a chemotaxis response ratio of 4.7 (±0.9) and 8.0 (±1.3) at 50 mg/L and 100 mg/L concentrations, respectively (Figure 2a). These results also show in the seawater bacterial consortium (Figure S1) i.e., high chemotactic responses (1.6 ± 0.4) of seawater bacterial consortium to the XG by 1 mg/L of concentration. However, HA showed the higher chemotactic response of 1.8 (±0.5) and 2.2 (±0.2) at 50 mg/L and 100 mg/L concentrations (Figure S1a).

We assumed that the greater electronegativity of organic compound particles might reduce bacterial movement to the particles, due to the presence of repulsive forces. However, we found no relationship between the surface charge of organic substance particles and the chemotactic response ratio of *P. aeruginosa* PAO1 (Figure 2b) and of the seawater bacterial consortium (Figure S1b). The different impacts between XG and HA on the chemotactic responses, although they have a similar electronegativity, might be caused by the different steric repulsion forces in a given condition. This indicates that the changes in electronegativity by deposition of organic substances might not impact on the initial bacterial attachment.

### 3.2. Bacterial Movement towards Membranes Pre-Conditioned with SA and BSA Organics

**Figure 3:**
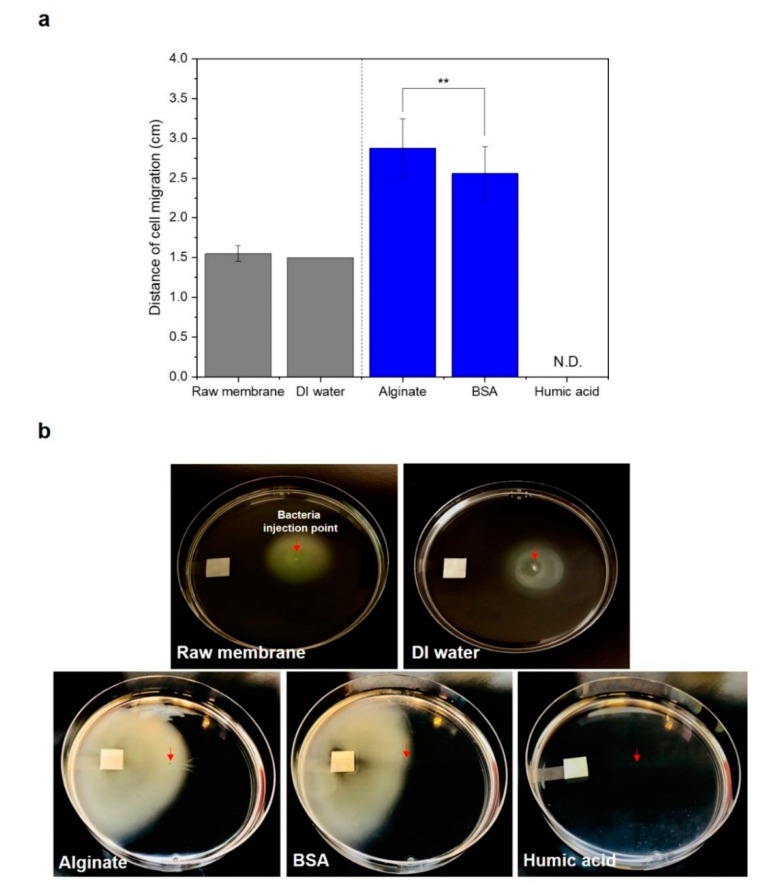
Migration of *P. aeruginosa* PAO1 on peptone semi-solid agar. (**a**) Migration distance from the bacteria injection point to the membrane was measured. Bacteria did not move in any direction heading to the membrane containing humic acid (HA) (N.D.: not detected). *P* values between sodium alginate (SA) and bovine serum albumin (BSA) conditions are calculated using a student’s *t*-test (0.05 < ** *p* < 0.5). (**b**) Photographs of semi-solid agar with bacterial migration. The red arrows indicate a bacterial injection point, and the membrane size was 1 × 1 cm^2^ (*n* = 7).

We observed swarming motility of *P. aeruginosa* PAO1 to the organic substances (100 mg/L)-conditioned cellulose filter membranes. The bacteria did not actively move towards raw membrane (without pre-conditioning) and pre-conditioned membrane with DI water. The bacteria were dispersed around the injection point with a distance of 1.5 cm (Figure 3). The distances of bacterial movement to the SA, BSA-conditioned membranes, after 24 h incubation, were 2.9 (±0.4) cm and 2.6 (±0.3) cm, whereas bacterial cells did not move towards the HA-conditioned membrane (Figure 3). As following the manufacturer, the molecular weight distributions were: HA 2–500 kDa, BSA ~66 kDa, and SA 120–190 kDa. Due to their molecular size being less than 2.5 µm, the organic substances could pass through the cellulose filter paper to the agar medium. The limited movement of bacteria to the HA showed that HA did not use a nutrient source for the bacteria. In the swarm plate, once bacteria were inoculated on the soft agar, they moved toward the concentration gradient of nutrients produced by metabolizing carbon nutrients [25,26]. Therefore, the bacteria could not swim in the absence of metabolizable compounds [26]. In the poor-nutrient condition, bacteria shut off motility to maintain energy and substrates [27].

The different *P. aeruginosa* PAO1 motility behavior towards HA in capillary and swarming agar might be due to the metabolism ability of HA by *P. aeruginosa* PAO1. In the capillary systems, a chemotaxis response can occur with the metabolism-independent chemoattractant [28], whereas bacterial motility in swarm agar is available only for the metabolizable compounds [26]. We assumed that the nutrient gradient could cause a strong chemotactic responses of *P. aeruginosa* PAO1 to the HA in the feed solution as shown in Figure 2a. However, results from the swarming motility test showed that HA was not used as a nutrient source of *P. aeruginosa* PAO1 (Figure 3). HA in the water impacts on the bacterial growth indirectly or directly. Indirectly, HA chelate toxic concentrations of metal ions such as Fe and Cu in the water, and supply the chelated essential cations that can facilitate bacterial growth [29]. HA can directly affect microbial metabolism by uptake of adequate molecular size and concentrations, or by mediating microbial respiration [29,30]. The concentrations of 10–100 mg/L and the lower molecular weight ranges (5.5–30 kDa) of humic fractions enhanced the microbial metabolism [31,32]. Therefore, the higher molecular weight sizes (2–500 kDa) of applied HA in this study might not be a favorable condition for uptake.

### 3.3. No Significant Relationships between Bacterial Attachment and Hydrophobicity or Surface Charge, but Exponential Relations with Membrane Roughness of Conditioned Membranes

Based on the bacterial chemotaxis response to the particles of the organic compound in the capillary system, HA attracted more bacteria (see Section 3.1). To verify the impact of the conditioning layer on the bacterial attachment in the membrane systems, we investigated the interaction between the layer conditioned by organic compounds and the bacteria. The impact of physicochemical properties of conditioning film including surface free energy, surface hydrophobicity, membrane roughness, and surface charge on initial bacterial attachment was investigated. Conditioning layers affect bacterial attachment by changes in the physicochemical properties of solid surfaces such as hydrophobicity, surface tension, surface charge, and roughness [6,33].

**Figure 4:**
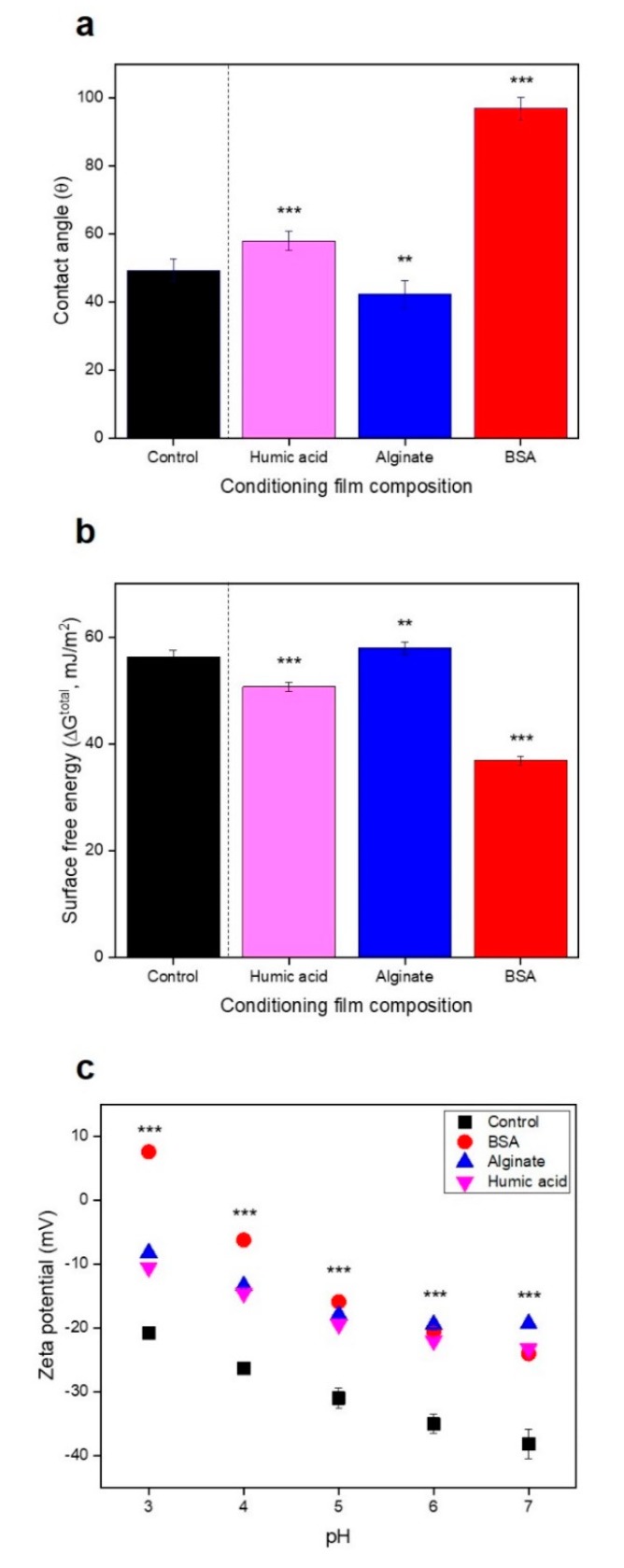
Characterization of conditioned reverse osmosis (RO) membranes: (**a**) Hydrophobicity, (**b**) surface free energy, and (**c**) surface charge, for pH ranging from 3 to 7 (*n* = 3). *P* values are calculated based on control (virgin RO) values, using student’s *t*-test (*** *p* ≤ 0.05, 0.05 < ** *p* < 0.5).

**Figure 5:**
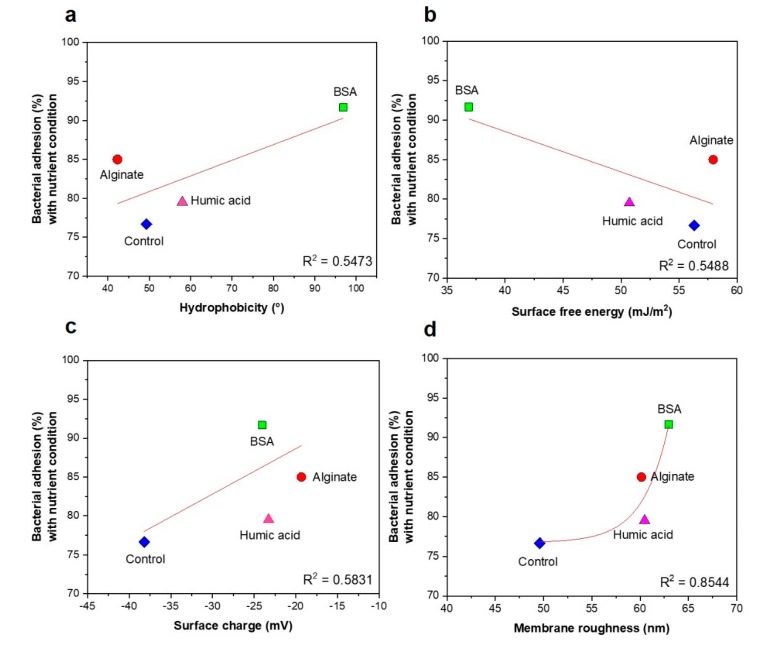
Relationships between the potential of bacterial attachment (%) with nutrients and (**a**) hydrophobicity, (**b**) surface free energy, (**c**) surface charge at pH 7, and (**d**) membrane roughness of organic-compounds-conditioned membranes. Control indicates bacterial attachment on the clean RO membrane, with no layer conditioned by organic compounds.

We found the hydrophobicity (wettability) of the virgin RO membrane (control) and conditioned membranes by HA, SA, and BSA to be 49.3 ± 3.3°, 58.0 ± 2.8°, 42.3 ± 4.1°, and 96.9 ± 3.3°, respectively (Figure 4a). The property of the hydrophobic BSA-conditioned membrane can be due to hydrophobic species such as alkyl and aromatic groups in BSA [6]. The hydrophobic lipopolysaccharides on the membrane of the bacterial cell will preferably bind to the hydrophobic surface rather than the hydrophilic surface [34]. Yuan et al. (2017) reported that the moderate membrane hydrophobicity (~90°) induced a high level of bacterial attachment due to the hydrophobic interactions between the bacterial membrane and the solid surface. However, the extremely hydrophilic (0°) or hydrophobic (>150°) surface of the membrane reduces the bacterial attachment [34]. Also, other studies showed that the super-hydrophobic membrane surface (or low surface free energy) promote bacterial attachment on the polymer surface by reducing the near-wall velocity of the bacteria [34,35]. In our study, we investigated the relationship between membrane hydrophobicity and bacterial attachment. The membrane hydrophobicity was shown in the order of BSA > HA > control > SA-conditioned membranes. In agreement with other studies, *P. aeruginosa* PAO1 preferred to attach on the hydrophobic BSA-conditioned membrane surface (91.7% attachment). However, no correlation between membrane hydrophobicity and bacterial attachment was observed (*R*^2^ = 0.5473) (Figure 5a).

The surface free energy of control, HA, SA, and BSA-conditioned RO membranes were 56.3 (±1.3), 50.7 (±0.9), 57.9 (±1.2), and 36.9 (±0.9) mJ/m^2^, respectively (Figure 4b, Table S2). In the initial bacterial attachment stage, they are required to overcome energy barriers such as Lifshitz-van der Waals attractive force, electrostatic repulsive force, and acid-base forces [36]. Therefore, the changes in surface free energy by different conditioning film compositions can affect bacterial attachment. The positive surface free energy showed the presence of hydrophilic interactions between the conditioned membranes and water [9]. The surface free energy has an inverse relationship with the bacterial attachment. However, no correlation with the bacterial attachment was observed (*R*^2^ = 0.5488), similar to the relation between hydrophobicity and bacterial attachment (Figure 5b).

Since RO and NF membranes have a negative charge in general, once bacteria approach the membranes, negative charged bacterial cells have a repulsive force to the membranes. Therefore, better attachment is shown to the hydrophobic membrane surfaces [37]. Zhao et al. (2015) found that the strong negative surface charge of NF membranes caused by HA and SA inhibited bacterial attachment [9]. For pH ranging from 3 to 7, the electronegativity of organic compounds-conditioned membranes was reduced, compared to the virgin RO membrane (−38.2 ± 2.3) (Figure 4c), indicating that the conditioning film may increase bacterial attachment. Figure 5c shows higher bacterial attachment on conditioned membranes than on virgin RO membrane. However, no correlation between bacterial attachment and surface charge at pH 7 was observed (*R*^2^ = 0.5831).

The membrane roughness (*S_q_*) and surface skewness (*S_sk_*) was observed under DI water, without nutrients (in PBS) and with nutrients (in M9 medium) conditions. The organic substances pre-conditioned membranes under DI water, the S_q_ of control, HA, SA, and BSA-conditioned RO membranes were 113.3 (±21.2), 153.0 (±53.6), 84.6 (±34.9), and 80.3 (±2.1) nm, respectively (Table 3, Figure S2). The factor of surface texture uniformity, the S_sk_ of these membrane coupons, were −0.4 (±0.3), 1.2 (±0.7), 1.1 (±0.4), and 1.0 (±0.2), respectively (Table 3). The S_sk_ value indicates asymmetry of height distribution; therefore, when the S_sk_ value is close to 0, it shows that the membrane has uniformity [38]. The changes in membrane roughness of the virgin RO membrane (control) was highly influenced by the feed solution compositions, while the organic pre-conditioned membranes’ roughness was not significantly changed (Table 3). The pre-deposited organic substances might affect the stability of the membrane roughness. The exponential relationship between the membrane roughness and the bacterial attachment was characterized by an exponential correlation coefficient (*R*^2^) of 0.8544 (Figure 5d). There are contradictory studies regarding the effects of membrane roughness on the initial bacterial attachment [8,33,36,39,40,41]. There was no clear correlations between surface roughness and the bacterial adhesion [39]. On the contrary, other studies [8,33,40,41] reported that bacteria preferably attached to the rougher and heterogeneous membranes with valleys for the bacterial deposition than to the smoother and homogenous membranes.

### 3.4. Nutrients in the Feed Solution Enhances Initial Bacterial Attachment

**Figure 6:**
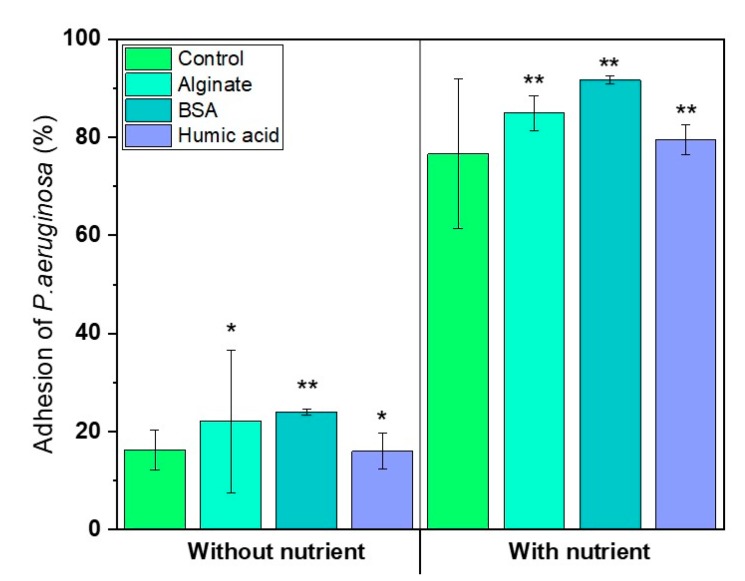
Impact of nutrients in the feed solution on bacterial attachment during 6 h. *P. aeruginosa* PAO1 inoculated in the solutions, either with or without nutrients. Nutrients in the feed solution positively affect bacterial attachment (*n* = 3). *P* values are calculated, in comparison with control values (virgin RO using student’s *t*-test (* *p* > 0.5, 0.05 < ** *p* < 0.5)).

Bacteria prefer to attach on the surface to take up nutrients in the nutrient-poor condition [42]. We hypothesized that the organic nutrient source from the conditioning film might enhance bacterial attachment in the absence of nutrients in the feed solution. So, the bacterial attachment on the conditioned-membranes was observed with or without nutrients in the feed solution. Figure 6 shows the bacterial attachment on the conditioned RO membranes, either with or without nutrients in the medium. No significant impact of conditioning film compositions on the bacterial attachment was observed in both cases (*p* > 0.05) (Figure 6). However, the result showed that the BSA-conditioned membrane attracted more bacteria than other conditioned-membranes. The bacterial attachment ability was 16.3% (±4.0%), 22.1% (±14.6%), 24.0% (±0.7%), and 16.0% (±3.7%) on control (without conditioning layer) for the SA, BSA, and HA-conditioned membranes, respectively, with no nutrients in the feed solution. In the presence of nutrients, bacterial attachment rate was 76.7% (±15.3%), 85.0% (±3.5%), 91.7% (±0.8%), and 79.5% (±3.1%) for the control, SA, BSA, and HA-conditioned membranes, respectively. The higher bacterial attachment in the nutrient condition (compared to the limited nutrient condition) was attributed to the microbial activity to move towards the membrane’s surface.

**Figure 7:**
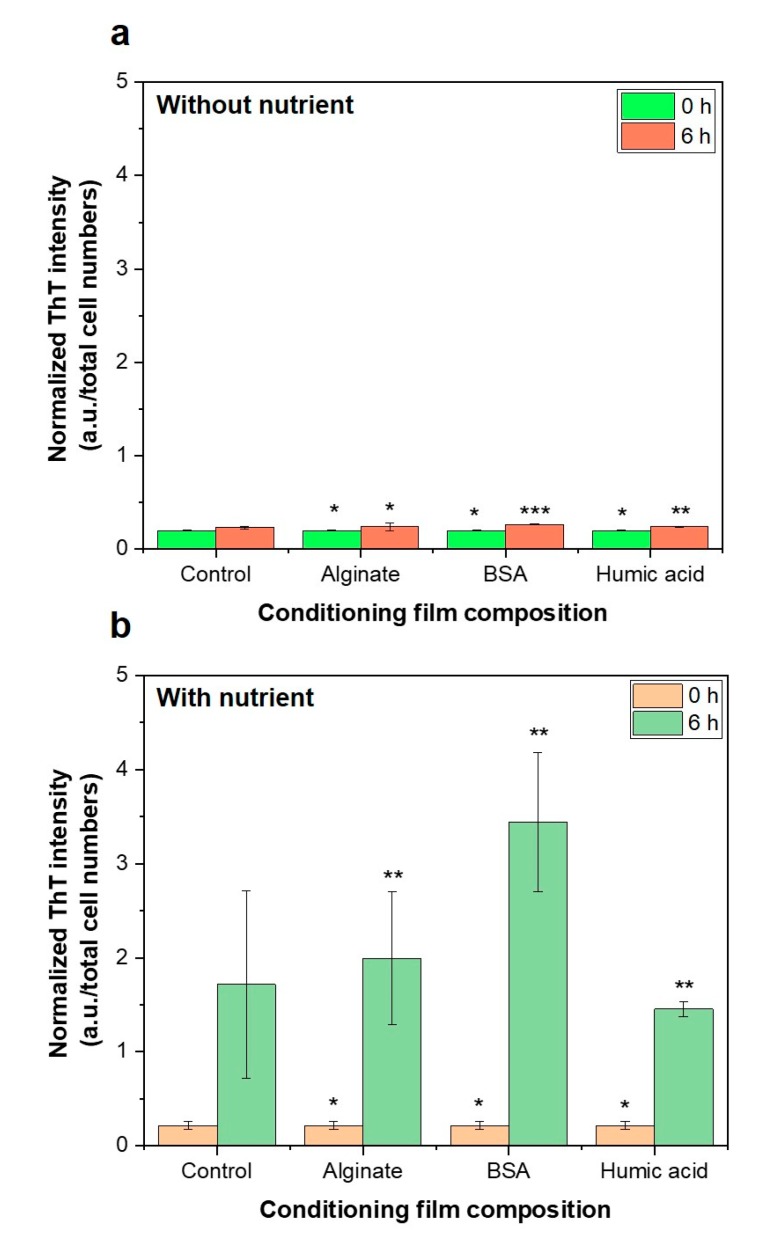
Normalized thioflavin T (ThT) fluorescence intensity of suspended bacterial cells for incubation times of 0 h and 6 h, (**a**) without and (**b**) with a nutrient. The higher ThT intensity indicates a higher potential of bacterial motility in the feed solution. *P* values were calculated compared to control sample (virgin RO) using student’s *t*-test (* *p* > 0.5, 0.05 < ** *p* < 0.5, *** *p* ≤ 0.05)).

The bacterial motility to the conditioned-membranes was investigated by staining bacteria with a ThT staining dye and measuring the fluorescence intensity (Figure 7). The ThT staining dye binds to the *β*-sheet of Fap fimbriae that modulates the attachment of *Pseudomonas* sp. to the abiotic surfaces [43]. Without nutrition in the medium, less bacterial motility was found, and the motility was not dependent on the type of conditioning film (Figure 7a). In the presence of nutrients in the feed solution, the bacterial motility was increased to 6 h incubation time, and more bacterial activity was observed in BSA-conditioned membranes (Figure 7b). This result indicates that BSA attracts more bacterial attachment by an increase in bacterial motility. We found that bacterial motility was inactivated in the absence of nutrients and that the bacterial motility potential varied depending on the composition of the conditioning film when using nutrients in the feed solution (*n* = 3).

In terms of membrane roughness after attachment of bacteria, in the presence of nutrients, bacterial attachment on the BSA-conditioned membrane was higher than in other conditions. This higher bacterial attachment on the BSA-conditioned membrane can be due to the high hydrophobicity and roughness of the membrane [9].

**Figure 8:**
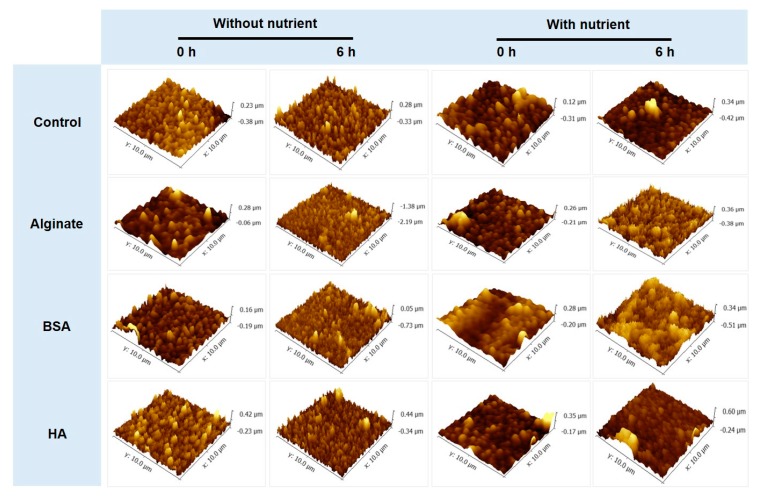
Atomic force microscopy (AFM) images of fouled membranes under the absence or presence of nutrients in the feed solution (scanned area, 10 × 10 µm^2^).

**Figure 9:**
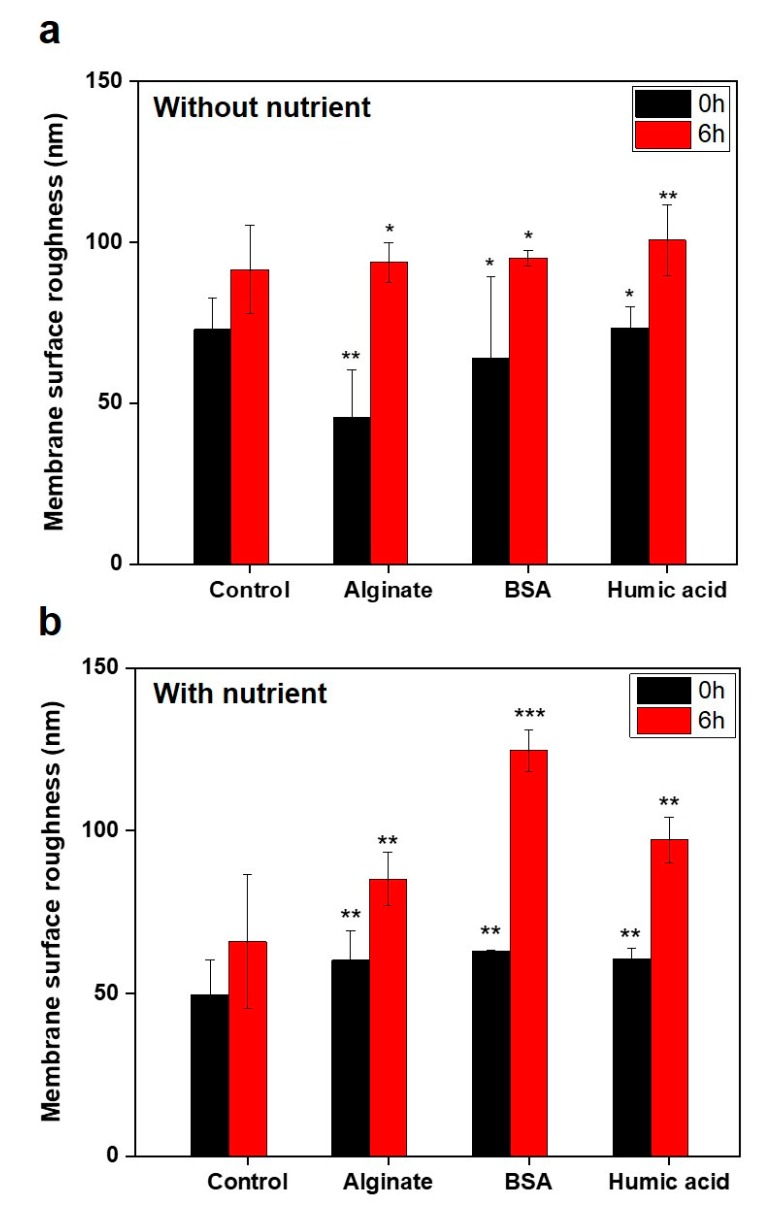
Membrane roughness of control and SA, BSA, and HA-conditioning layers after incubation with *P. aeruginosa* PAO1 for 6 h, either (**a**) without or (**b**) with nutrient conditions (*n* = 3). *P* values are calculated based on the values obtained for the control (virgin RO) film using student’s *t*-test (* *p* > 0.5, 0.05 < ** *p* < 0.5, *** *p* ≤ 0.05).

We analyzed the changes in membrane roughness before and after incubation with the *P. aeruginosa* PAO1 formation on the pre-conditioned membranes with or without nutrients in in the feed solutions (Figure 8, Figure 9, Table S3). After 6 h incubation, in the absence of nutrients, the HA-conditioned membrane exhibited a higher roughness (100.6 nm) compared to control (69.7 nm), SA (93.7 nm), or BSA (95.1 nm)-conditioned membranes (Figure 9a). In the presence of nutrients, we observed a higher roughness on the BSA (124.6 nm) and HA (97.2 nm)-conditioning film layers compared to the control (65.9 nm) and SA (81.2 nm) (Figure 9b). The deposited membrane surfaces of pre-conditioned membranes after 6 h incubation were clearly shown in Figure S3. The increases in the membrane roughness might be caused by the attachment of bacteria as well as deposition of extracellular polymeric substances (e.g., polysaccharides, proteins). Results showed that HA and BSA-conditioned membranes might have a higher potential for biofilm formation than SA or control samples. We conclude that the nutrient components in the feed solution can be a significant factor in inducing bacterial growth and the formation of a biofilm.

### 3.5. Importance of Bacterial Metabolic Activity on the Initial Bacterial Attachment

In this study, to inhibit bacterial activity (or motility) without changes in the structure of the bacterial surface (such as flagella or pili), bacterial cells were pretreated with glutaraldehyde [24]. We hypothesized that the loss of bacterial activity might inhibit attachment on the surface of the membrane, regardless of the composition of the conditioning layer.

**Figure 10:**
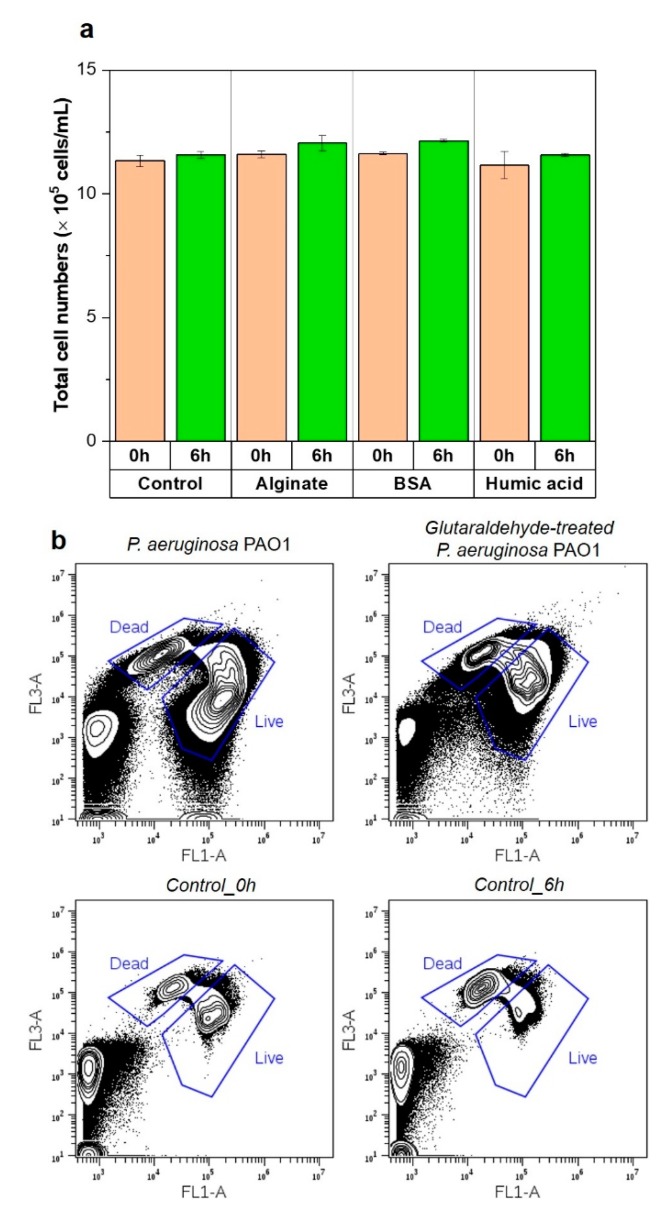
(**a**) Attachment of damaged bacterial cells treated by glutaraldehyde on conditioned membranes composed of control (without organic conditioning layer), sodium alginate (SA), bovine serum albumin (BSA) and humic acid (HA). Suspended bacteria were taken at 0 h and 6 h (*n* = 3). (**b**) Flow cytometry density plots of *P. aeruginosa* PAO1 (100 times dilution), glutaraldehyde-treated *P. aeruginosa* PAO1 (100 times dilution) and incubation control samples at 0 h and 6 h. The gating area indicates live and dead bacterial cells, respectively.

Results showed no changes in the number of bacterial cells in the feed solution observed after a 6 h incubation (Figure 10a), in contrast with the results obtained with a bacterial attachment test without nutrients (~20% of attachment rate) (Figure 6). No changes in bacterial cell numbers in the feed solution indicates the glutaraldehyde-treated bacterial cells did not attach to the surface of the membrane surface with the loss of bacterial metabolic activity. Also, the cell viability of glutaraldehyde-treated *P. aeruginosa* PAO1 at 0 h (54.3 ± 0.5) was reduced at 6 h (12.3 ± 0.4) (Figure 10b). Many studies [12,44,45] illustrated the role of bacterial motility on the initial bacterial attachment. The initial attachment is not entirely reduced by the elimination of flagella from the bacteria, and regardless of bacterial motility, environmental conditions such as nutrient concentration and hydraulic water flow conditions may significantly affect bacterial attachment [45]. This study also shows that the filaments (i.e., flagella and pili) of *P. aeruginosa* PAO1 had no impact on the initial bacterial attachment with loss of the microbial metabolic activity.

In the cross-flow membrane systems, the deposition of particles on the virgin membranes occurs in a short period (within minutes) [46], and bacteria are attached subsequently, which leads to decreases in permeate flux. In this stage, various forces, including hydrodynamic drag forces, concentration of polarization, and cross-flow, play an important role in the bacterial attachment and development of biofilm [8,47]. This study showed that the bacterial chemotactic forces did not have a significant impact on bacterial attachment. Instead, the nutrients in the feed solution and microbial metabolic activity strongly affect the bacterial attachment. However, the importance of microbial metabolic activity, which is related to bacterial motility, could be reduced in the cross-flow systems since the permeate drag force can affect the deposition of bacteria regardless of the microbial metabolic activity [47]. Nevertheless, the deposition of active bacteria on the membrane surface can cause severe biofilm development by cell-to-cell communications (e.g., quorum sensing). In future studies, we plan to investigate the relationships between the composition of the conditioning film or concentrations and cell-to-cell communication in the feed solution (or attached bacteria). Understanding the role of conditioning films on surfaces on the initial and later stage formation of the biofilm opens new doors on control strategies in membrane systems for seawater desalination and wastewater reuse.

## 4. Conclusions

This study investigated bacterial responses to the organic substances that form the conditioning film on the membrane. Different chemotactic responses of *P. aeruginosa* PAO1 to the particles of organic compounds (in a bulk solution) in RO membranes were observed.

The key findings of this study are:

- Chemotactic response of *P. aeruginosa* PAO1 to the organic substances did not influence bacterial attachment on organic pre-conditioned RO membranes.
- Changes of membrane roughness by a conditioning film affected bacterial attachment, whereas hydrophobicity, membrane surface charge and surface free energy did not affect bacterial attachment.
- Presence of nutrients and microbial metabolic activity in the feed solution showed a significant impact on initial bacterial attachment.

## Figures and Tables

**Table 1:** Impact of conditioned membranes on bacterial attachment and biofilm formation.

Composition of the Conditioning Film	Substratum	Bacteria	Key Findings	Ref.
^a^ SA, BSA	NF membrane (DOW Filmtec, USA)	*Pseudomonas putida*	Higher electron-donor functionality and membrane roughness by organic fouling enhanced bacterial attachment.	[8]
SA, BSA, HA	NF membrane (Synder, USA)	*Pseudomonas aeruginosa*	Smooth, hydrophilic, negative surface charged membranes conditioned by SA and HA-calcium inhibited bacterial attachment. Rough and hydrophobic conditioned membrane caused by BSA-calcium enhanced bacterial attachment.	[9]
Synthetic wastewater	RO membrane (DOW Filmtec, USA)	*Pseudomonas aeruginosa*	Biofouling on conditioned membrane strongly impacts on permeate flux and salt rejection than biofouling on the virgin membrane.	[10]
HA, AA	NF membrane (DOW Filmtec, USA)	*Pseudomonas fluorescens*	Bacterial attachment to the organic-compounds-conditioned membranes depends on the thickness of conditioning layers.	[11]
BSA, SA, NOM	Ultrafiltration (UF) membrane (GE Osmonics, USA)	*Escherichia coli*	Ionic strength, pH, and calcium ion concentrations of feed solution affect bacterial attachment.	[12]
AA, BSA	RO membrane (Toray, Japan)	*Pseudomonas aeruginosa* PAO1	Greater bacterial attachment with the increase of conditioning layer coverage.	[13]
AA	RO membrane (Hydranuatics, PA)	*Pseudomonas aeruginosa* PA14	Correlation between transparent exopolymer particles (TEP) concentration and initial bacterial deposition on the RO membranes.	[14]

^a^ Abbreviation: Bovine serum albumin (BSA), humic acid (HA), sodium alginate (SA), alginic acid (AA), natural organic matter (NOM), reverse osmosis (RO).

**Table 2:** Summary of key issues being investigated.

Title	Experimental Scheme	Analysis	Matrix	Results
2.1. Chemotaxis responses of *P. aeruginosa* PAO1 to the organic substances in the feed solution	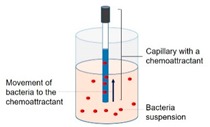	Capillary test	Liquid solution	Figure 2
2.2. Swarming behavior of *P. aeruginosa* PAO1 on swarm plates	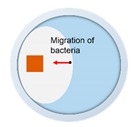	Swarming motility	Swarm plates	Figure 3
2.3. Impact of physicochemical properties of organic pre-conditioned membranes on bacterial attachment	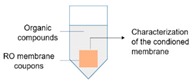	Characterization of membrane roughness, hydrophobicity, surface charge	RO membrane	Table 3, Figures 4 and 5
2.4. Impact of nutrients in the feed solution on bacterial attachment	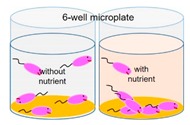	Quantification of total cell numbers and bacterial motility in the feed solution		Figures 6–9
2.5. Impact of microbial activity in the feed solution on bacterial attachment	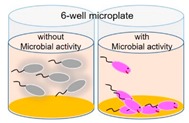			Figure 10

**Table 3:** Membrane roughness and skewness of conditioning layers on RO membranes under control (in deionized (DI) water), without (in phosphate-buffered saline (PBS)) and with nutrients (in M9 medium) conditions.

Composition of the Conditioning Film	Control (in DI Water)		Without Nutrients (in PBS)		With Nutrients (in M9 Medium)	
	Roughness (S_q_) (nm)	Skewness (*S_sk_*)	Roughness (S_q_) (nm)	Skewness (*S_sk_*)	Roughness (S_q_) (nm)	Skewness (*S_sk_*)
Control	113.3 (±21.2)	−0.4 (±0.3)	72.9 (±9.7)	0.9 (±0.6)	43.9 (±5.4)	0.9 (±0.1)
Humic acid	153.0 (±53.6)	1.2 (±0.7)	73.4 (±6.4)	1.6 (±0.6)	60.5 (±3.3)	1.9 (±1.1)
Alginate	84.6 (±34.9)	1.1 (±0.4)	45.5 (±15.0)	1.4 (±0.8)	60.1 (±9.2)	1.7 (±0.4)
BSA	80.3 (±2.1)	1.0 (±0.2)	64.0 (±25.2)	1.2 (±0.2)	63.0 (±0.4)	0.4 (±0.3)

## Supplementary Materials

The following are available online at https://www.mdpi.com/2077-0375/9/12/162/s1. Chemotactic responses of seawater mixed consortium to the organic substances and relations with surface charge of organic substances (Figure S1), three-dimensional AFM images of organic substance (control, alginate, BSA, HA) pre-conditioned RO membranes (Figure S2), AFM images of control (virgin RO membrane) and pre-conditioned membranes by alginate, BSA, and HA at 0 h and 6 h incubation with P. aeruginosa PAO1 under with nutrient condition (Figure S3), composition of M9 medium (Table S1), the surface energy of organic pre-conditioned RO membranes (Table S2), and membrane roughness and skewness of biofilm formed-conditioned membranes (Table S3) are provided.
