# Supplementary material for: Insignificant Impact of Chemotactic Responses of Pseudomonas aeruginosa on the Bacterial Attachment to Organic Pre-Conditioned RO Membranes
Source: Membranes (Basel). 2019 Dec 2;9(12):162. doi: 10.3390/membranes9120162 (PMC6950137; doi:10.3390/membranes9120162)
Supplement: Supplementary file 1 [file membranes-09-00162-s001.pdf]

Supplementary Information

Insignificant Impact of Chemotactic Responses of *Pseudomonas aeruginosa* on the Bacterial Attachment to Organic Pre-Conditioned RO Membranes

Lan Hee Kim[1] and Johannes S. Vrouwenvelder[1,2,*]

[1] Water Desalination and Reuse Center (WDRC), Division of Biological and Environmental Science and Engineering (BESE), King Abdullah University of Science and Technology (KAUST), Thuwal 23955-6900, Saudi Arabia; lanhee.kim@kaust.edu.sa
[2] Department of Biotechnology, Faculty of Applied Sciences, Delft University of Technology, Van der Maasweg 9, 2629 HZ Delft, The Netherlands
* Correspondence: johannes.vrouwenvelder@kaust.edu.sa

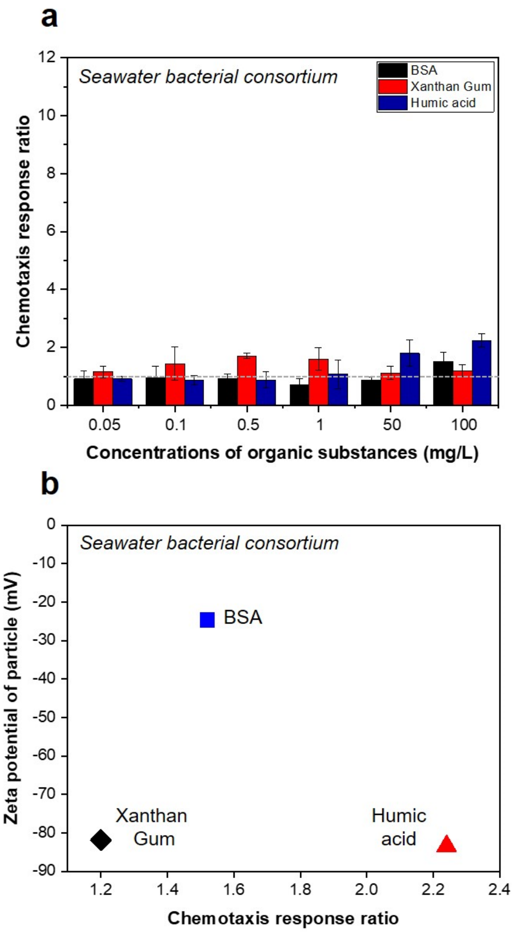

Figure S1. (**a**) Chemotactic responses of seawater bacteria to the organic substances such as BSA, xanthan gum, and humic acid. The dotted line indicate the chemotaxis response ratio of "1". (**b**) Relations between surface charge of organic particles and chemotaxis response ratio.

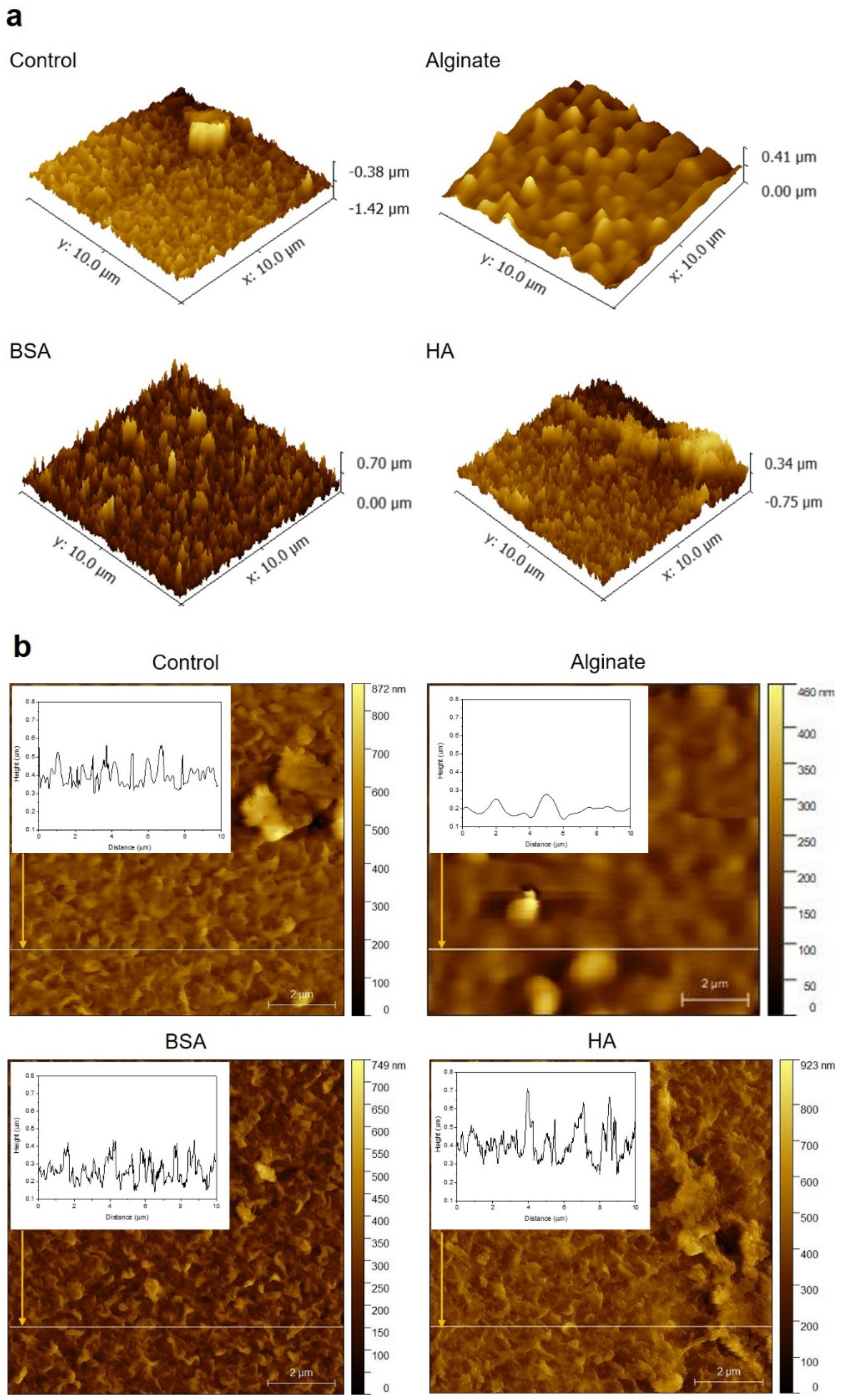

Figure S2. (a) Three-dimensional AFM images of organic substance (control, alginate, BSA, HA) pre-conditioned RO membranes with scanned areas of 10 × 10 μm^2. (b) Height profiles along the lines (yellow line).

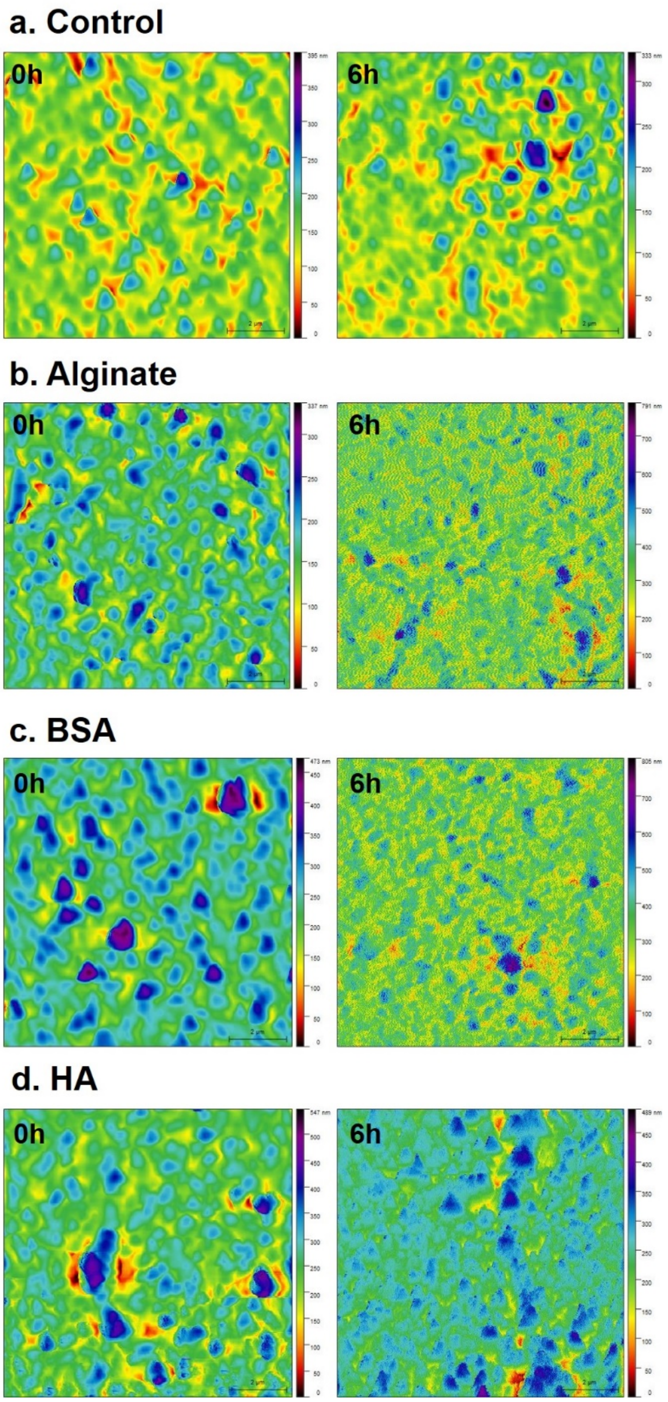

Figure S3. AFM images of (**a**) control (virgin RO membrane) and pre-conditioned membranes by (**b**) alginate, (**c**) BSA, and (**d**) HA at 0 h and 6 h incubation with *P. aeruginosa* PAO1. The significant spectral color changes were shown in the organic substances pre-conditioned membranes compared to the control after 6 h incubation.

Table S1. Composition of M9 medium.

Composition	Volume (mL)
M9 salts [a]	200
1 M $MgSO_4$	2
20% glucose	20
1 M $CaCl_2$	0.1
Total	1000

[a] M9 salts (g/L): 64, $Na_2HPO_4 \cdot 7H_2O$; 15, KH_2PO_4; 2.5, NaCl; 5.0, NH_4Cl

Table S2. Surface energy of organic pre-conditioned RO membranes

**Membrane Coupon**	**Contact Angle (θ)**			**Surface Energy (mJ/m^2)**		
	**Water**	**Formamide**	**Diiodomethane**	γ_s^d	γ_s^p	γ_s
Virgin RO	49.31 (±3.25)	16.99 (±1.79)	24.86 (±3.13)	34.44 (±0.74)	21.86 (±0.50)	56.29 (±1.25)
Alginate	42.3 (±4.06)	25.93 (±7.30)	27.71 (±2.47)	31.63 (±0.58)	26.31 (±0.59)	57.94 (±1.18)
BSA	96.86 (±3.33)	69.57 (±3.71)	44.46 (±2.23)	33.74 (±0.59)	3.13 (±0.28)	36.87 (±0.87)
Humic acid	57.99 (±2.76)	34.14 (±3.29)	28.88 (±2.18)	34.53 (±0.53)	16.18 (±0.34)	50.71 (±0.87)

γ_s^d, dispersion component; γ_s^p, polar component; γ_s; surface free energy

Table S3. Membrane roughness and skewness of biofilm formed-conditioned membranes

**Composition of conditioning film**	Incubation time (h)	Membrane roughness (S_q, nm)		Skewness (S_{sk})	
		Without nutrient	With nutrient	Without nutrient	With nutrient
**Control**[a]	0	72.9 (±9.7)	43.9 (±5.4)	0.9 (±0.6)	0.9 (±0.1)
	6	69.7 (±9.1)	65.9 (±20.6)	1.2 (±0.6)	1.8 (±1.5)
	48	72.8 (±2.4)	44.8 (±7.7)	0.8 (±0.1)	0.6 (±0.4)
Alginate	0	45.5 (±15.0)	60.1 (±9.2)	1.4 (±0.8)	1.7 (±0.4)
	6	93.7 (±6.1)	81.2 (±6.2)	0.8 (±0.1)	0.5 (±0.1)
	48	66.3 (±4.5)	55.8 (±5.3)	0.7 (±0.1)	1.0 (±0.0)
BSA	0	64.0 (±25.2)	63.0 (±0.4)	1.2 (±0.2)	0.4 (±0.3)
	6	95.1 (±2.5)	124.6 (±6.5)	0.6 (±0.1)	0.3 (±0.0)
	48	106.2 (±17.7)	124.8 (±31.3)	2.6 (±0.6)	1.9 (±0.8)
Humic acid	0	73.4 (±6.4)	60.5 (± .3)	1.6 (±0.6)	1.9 (±1.1)
	6	100.6 (±11.0)	97.2 (±6.9)	1.0 (±0.4)	1.5 (±0.7)
	48	149.2 (±12.9)	90.0 (±13.2)	−0.4 (±0.0)	0.9 (±0.6)

[a] Without addition of organic substances
